# Mesenchymal stromal cell extracellular vesicles for multiple sclerosis in preclinical rodent models: A meta-analysis

**DOI:** 10.3389/fimmu.2022.972247

**Published:** 2022-11-04

**Authors:** Chengfeng Xun, Huiyin Deng, Jing Zhao, Lite Ge, Zhiping Hu

**Affiliations:** ^1^ Department of Neurology, Second Xiangya Hospital, Central South University, Changsha, China; ^2^ Hunan Provincical Key Laboratory of Neurorestoratology, the Second Affiliated Hospital, Hunan Normal University, Changsha, China; ^3^ The National & Local Joint Engineering Laboratory of Animal Peptide Drug Development, College of Life Sciences, Hunan Normal University, Changsha, China; ^4^ Department of Anesthesiology, the Third Xiangya Hospital, Central South University, Changsha, Hunan, China

**Keywords:** exosomes, extracellular vesicles, stem cells, multiple sclerosis, animal model, meta-analysis, systematic review

## Abstract

**Introduction:**

Extracellular vesicles (EVs), especially mesenchymal stem (stromal) cell-derived EVs (MSC-EVs), have gained attention as potential novel treatments for multiple sclerosis (MS). However, their effects remain incompletely understood. Thus, the purpose of this meta-analysis was to systematically review the efficacy of MSC-EVs in preclinical rodent models of MS.

**Methods:**

We searched PubMed, EMBASE, and the Web of Science databases up to August 2021 for studies that reported the treatment effects of MSC-EVs in rodent MS models. The clinical score was extracted as an outcome. Articles were peer-reviewed by two authors based on the inclusion and exclusion criteria. This meta-analysis was conducted using Stata 15.1 and R.

**Results:**

A total of twelve animal studies met the inclusion criteria. In our study, the MSC-EVs had a positive overall effect on the clinical score with a standardized mean difference (SMD) of -2.17 (95% confidence interval (CI)):-3.99 to -0.34, P = 0.01). A significant amount of heterogeneity was observed among the studies.

**Conclusions:**

This meta-analysis suggests that transplantation of MSC-EVs in MS rodent models improved functional recovery. Additionally, we identified several critical knowledge gaps, such as insufficient standardized dosage units and uncertainty regarding the optimal dose of MSC-EVs transplantation in MS. These gaps must be addressed before clinical trials can begin with MSC-EVs.

## Introduction

Disease of multiple sclerosis (MS) is a chronic and severe autoimmune demyelinating disease that primarily affects young adults in their early 20s and increasingly decreases their quality of life (1) The disease affects approximately 3 million people worldwide, presenting a significant health burden to the global community (2). As a result of its similarity to both clinical and pathological features of MS patients, the experimental autoimmune encephalomyelitis (EAE) animal model is one of the most widely used in MS research (3). Currently, therapeutic approaches focus on treating acute attacks and improving symptoms. There are several disease-modifying therapies available that alter the immune system, exerting anti-inflammatory activity and reducing the frequency of relapses, which can stabilize or delay the progression of disability or on occasion improve it (4). Even though over the past decade, significant progress has been made in the treatment of MS, the current therapeutic approaches remain limited. Consequently, there is an urgent need for novel types of drugs or therapies.

New treatments are necessary, and stem cell therapy is gaining momentum as an option. Many types of stem cells can be used, such as hematopoietic stem cells (5), however, mesenchymal stem (stromal) cells (MSCs) seem to be the most promising. MSCs are a type of multipotent cell with tremendous potential in biomedicine, especially in immunoregulation and tissue regeneration (6, 7). Several preclinical studies have demonstrated that MSC-based therapy is potentially effective in treating EAE by attenuating tissue injury and promoting tissue regeneration in laboratory animals. Unfortunately, only a small proportion of MSCs injected into injured tissues were recovered from intravenous injection. This led to a paradigm shift, in which MSCs were seen as a paracrine rather than a cellular factor. The extracellular vesicles (EVs) is a membrane-enclosed small vesicle with a diameter of 30-120 nm that can contain proteins, lipids, or microRNAs from the parent cells (8). Recent evidence indicates that MSC-EVs play an influential role in stem cell therapy, primarily by acting through a paracrine mechanism (9). Due to their low immunogenicity and ability to remain stable in circulation in contrast with stem cells, MSC-EVs have attracted significant attention from clinicians and researchers for their excellent efficacy and safety (10).

A systematic review is a type of literature review that aims to address a specific research question by gathering, selecting, analyzing, and synthesizing all the relevant evidence (11, 12). In contrast with traditional reviews, systematic reviews based on scientific methods can objectively evaluate all the current relevant research evidence and provide a more accurate assessment of results, which is regarded as the highest level of scientific evidence quality of the research being conducted (13, 14). Despite a certain number of animal studies have been performed to investigate the efficacy of MSC-EVs on an MS with various cell origins and different injection doses, delivery routes, and therapy times, there is still a lack of evidence-based research on this topic. To provide the most recent available evidence for clinical studies, we performed this meta-analysis to investigate the efficacy of MSC-EVs on preclinical rodent models.

## Materials and methods

### Search strategy and selection criteria

Preferred Reporting Items for Systematic Reviews and Meta-Analyses (PRISMA) reporting guidelines were followed in the conduct of the study (15). Our search strategy included using PubMed, Embase, and Web of Science databases as well as: searching with terms such as “mesenchymal stem cells” OR “mesenchymal stromal cells” OR “mesenchymal stem cell” OR “mesenchymal stromal cell”) AND (“Extracellular Vesicles” OR “Exovesicles” OR “Exosomes” OR “Endosomes”) AND (“Multiple sclerosis” OR ”MS” OR ”Experimental Autoimmune Encephalomyelitis” OR “ Experimental Allergic Encephalomyelitis “) (Additional file 1 for details). Additionally, the reference lists of eligible studies were reviewed to identify additional relevant publications. Analyzing published data does not require ethical approval or consent of the patient; the data is presented both in the original article and in the supplement to it. According to the database, the article was last searched on August 31, 2021, and only English was the language of publication.

This study used the PICOS scheme (population, intervention, control, outcome, and study design) to determine eligibility for participation (16). For the purposes of this meta-analysis, the following studies met the inclusion criteria: (a) The findings should be written and presented in English. (b) The research focused primarily on animal models of MS. (c) They evaluated the efficacy of SC-EVs treatment in animal models of multiple sclerosis (all types of animals of both sexes). (d) The studies provided adequate information regarding the functional outcome. It is imperative that the SC-EVs studied meet the standards of international guidelines for investigating EVs, which were published in 2018 and are entitled “Minimum Information for Studies of EVs” (MISEV 2018) (17). (e) Report experimental results in original scientific publications. In the case of two or more articles with overlapping information, we select the most recent or most informative of the two. The following studies are not considered: (a) participants in study groups who did not receive SC-EVs or those in which SC-EVs were not administered directly to the animals were excluded; (b) studies that did not involve *in vivo* testing; (c) the clinical outcomes were not reported; (d) SC-EVs were given before animal induction; and (e) articles of review, organizational guidelines, expert opinion articles, conference abstracts, or editorial correspondence without original data.

### Data abstraction

In the eligible studies, the following information was extracted and documented independently by two investigators: (a) general information (first author, publication year, and country); (b) experimental methods (number of animals per group for individual comparisons); (c) species and strain of animals; (d) gender; methods of MS induction in the animal model; (e) sources and types of MSCs; (f) dose of SC-EVs; (g) delivery route of MSCs; (h) method of extracting SC-EVs; (i) unit of dosage for SC-EV transplantation; (j) time of administration; (k) duration of follow-up; and (l) clinical score.

Using GetData Graph Digitizer software, values were calculated from images if only graphs were available. As part of the analysis, the two researchers’ readings were averaged. Whenever the standard deviation was not available, the standard error was converted to a standard deviation by multiplying it by the square root of the group size. When multiple experimental groups differed from the control group by a variety of factors, like EVs dose, delivery route, and timing, these groups were considered independent studies. Only the longest period was considered when results from a range of follow-up periods were evaluated.

### Statistical analysis

The primary outcome of this meta-analysis was mortality. standardized mean differences (SMDs) was calculated between the SC-EVs treated group and the control group to determine the combined effect size. All statistical analyses and graphs were performed by Stata 15.1 (StataCorp, College Station, TX, USA) (18), R language (version 4.1.3, www.r-project.org) and the meta package (version 5.2-0), using a random-effects model and the Hedges calculation (19). To display the pooled mean difference, we generated forest plots based on the SMD and 95% confidence interval of each study. It was considered significant if the P-value was smaller than 0.05. There was a random-effects model used if there was substantial heterogeneity (*I* ^2^ > 50%, p < 0.05) (20). We conducted sensitivity analyses to eliminate extreme values that may have contributed to the overall effect (21).

We used seven clinical characteristics to group the effect size of outcome: (a) gender (male, or female); (b) species (rat, or mouse); (c) MSCs types (adipose tissue-derived stem cells (ADSCs), bone marrow mesenchymal stem cell (BMSCs), umbilical cord mesenchymal stem cells (UCMSCs), or periodontal ligament stem cells (PDLSCs)); (d) MSCs species (Allogeneic, Syngeneic, or Xenogeneic); (e) extraction method of exosomes (differential centrifugation, or kit); (f) Time of delivery post MS induction (<14 days, or ≥14days); delivery route (intravenous (IV), or intranasal administration (IN)). In order to examine the possible associations between the outcomes and the above clinical characteristics, subgroup analyses and meta-regression analyses were performed (22). Publication bias was evaluated using funnel plots, and Egger regressions were used to assess the symmetry of funnel plots (23). The Trim-and-Fill method would be employed to correct any non-negligible bias (24).

## Results

### Identified and eligible studies

**Figure 1:**
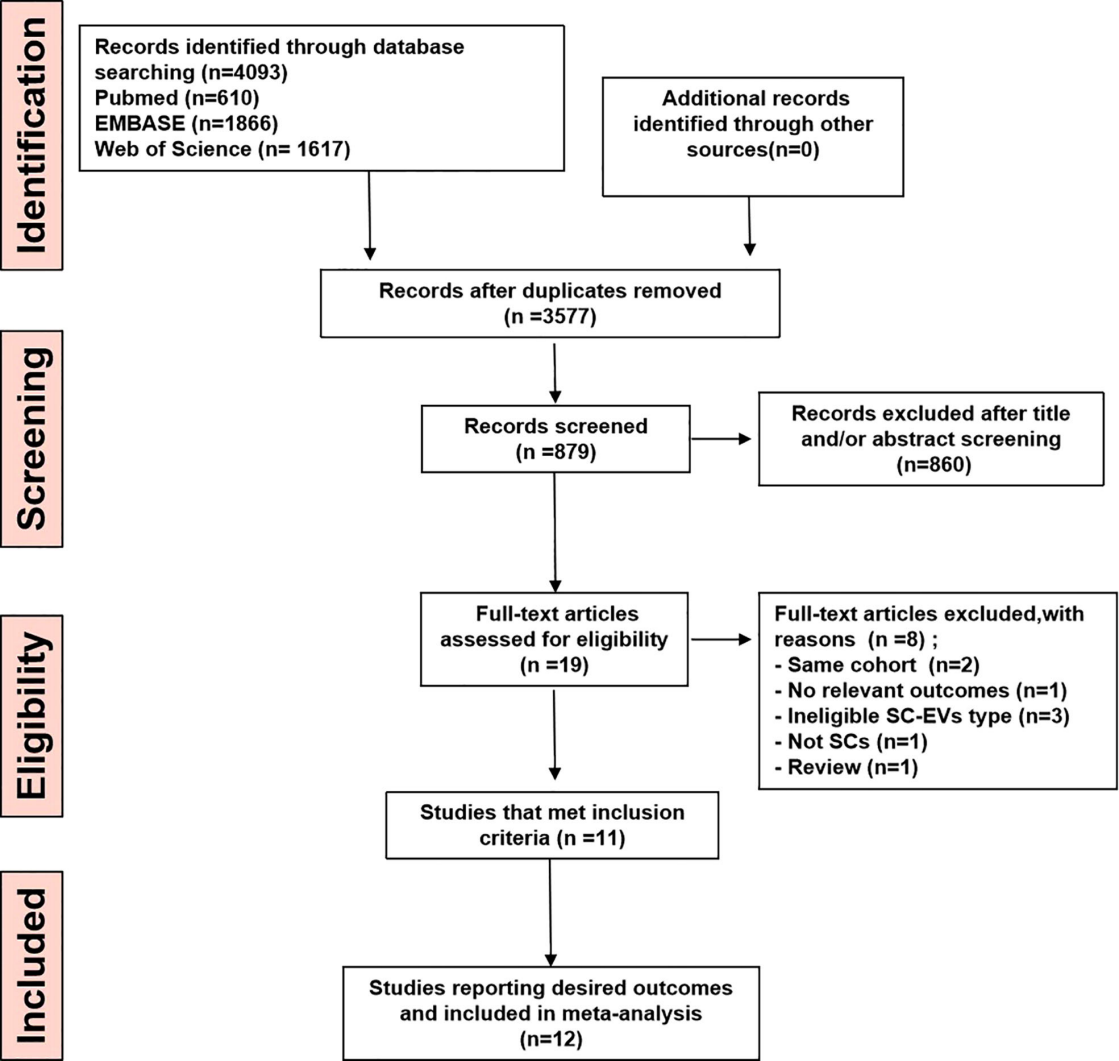
PRISM Aflow diagram for review and selection process of studies included in meta-analysis of MSC-EVs in rodent models of MS.

Literature searching identified 4,093 potential studies at the primary retrieval: 610 records in PubMed, 1866 records in Embase, and 1617 in Web of Science. A total of 874 full-text articles remained after the review and exclusion process. In addition, 860 records were excluded based on the reasons in **Figure 1**. Following an examination of the full texts of the remaining 19 articles, 7 were eliminated. Finally, data from 12 studies published between 2017 and 2021 contributed to the meta-analysis.

### Study characteristics

As shown in **Table 1**, the study characteristics are summarized (10, 25–32). All the research was carried out on rats and mice, and in most of the cases, the animals were female. In this animal model, MS was induced by administering myelin oligodendrocyte glycoprotein (MOG) or guinea pig spinal cord homogenate (GPSCH), with MOG accounting for the majority of incidences. The vast majority of studies used BMSCs and ADSCs, PDLSCs, or UC-MSC derived from mice, rats, or humans. Ultracentrifugation is the most common method of EV separation, though commercial kits may also be used. MSC-EVs were characterized by quantification, size distribution, morphological analysis, and/or surface marker expression in most studies. The units applied for dosing MSC-EVs varied considerably and included absolute protein amount and particle number. Additionally, the therapeutic dose of MSC-EVs varies greatly among the articles with absolute protein quantification as the unit, ranging between 5-400ug. An intravenous injection of MSC-EVs was typically used in most cases. Half of the studies involved a single transplant, and half involved two to three transplants. Further, MSC-EVs were administered from 0 days to 13 weeks following MS induction, with follow-ups ranging from 18 to 40 days.

**Table 1:** Summary of study characteristics of all included articles.

number	title	Author year Country	Species	Strain	Gender	the animal model of MS	stem cell type	stem cell source	Compatibility MSC-EVs Dose	Extraction method of exosomes	Time of delivery post-sepsis induction	MSC route	follow up
1	Human periodontal ligament stem cells secretome from multiple sclerosis patients suppresses NALP3 inflammasome activation in experimental autoimmune encephalomyelitis	Rajan et al. (2017)Italy (25)	mice	C57Bl/6	male	EAE(MOG)	PDLSC	Xenogenic	24 μg	Kit	at day 14	IV	28 days
2	Nanovesicles from adipose-derived mesenchymal stem cells inhibit T lymphocyte trafficking and ameliorate chronic experimental autoimmune encephalomyelitis	Farinazzo1 et al. (2018)Italy (26)	mice	C57Bl/6	not reported	EAE(MOG)	ADMSC	Allogeneic	5 μg	Differential centrifugation	at 3, 8 and 13 days	IV	36 days
3	Nanovesicles from adipose-derived mesenchymal stem cells inhibit T lymphocyte trafficking and ameliorate chronic experimental autoimmune encephalomyelitis	Farinazzo1 et al.(2018)Italy (26)	mice	C57Bl/6	not reported	EAE(MOG)	ADMSC	Allogeneic	5 μg	Differential centrifugation	at 12, 16 and 20 days	IV	36 days
4	Exosomes derived from mesenchymal stem cells attenuate inflammation and demyelination of the central nervous system in EAE rats by regulating the polarization of microglia	Li et al.(2019)A China (27)	rats	SD	female	EAE(GPSCH)	BMSC	Allogeneic	100μg	Differential centrifugation	day 0	IV	14 days
5	Exosomes derived from mesenchymal stem cells attenuate inflammation and demyelination of the central nervous system in EAE rats by regulating the polarization of microglia	Li et al.(2019)B China (27)	rats	SD	female	EAE(MOG)	BMSC	Allogeneic	400μg	Differential centrifugation	day 0	IV	14 days
6	Immunomodulatory properties of MSC-derived exosomes armed with high affinity aptamer toward mylein as a platform for reducing multiple sclerosis clinical score	Shamili et al.(2019)Iran (28)	mice	C57Bl/6	female	EAE(MOG)	BMSC	Allogeneic	200μg	Kit	at 1, 3, 6 days	IV	28 days
7	Immunomodulatory properties of MSC-derived exosomes armed with high affinity aptamer toward mylein as a platform for reducing multiple sclerosis clinical score	Shamili et al.(2019)Iran (28)	mice	C57Bl/6	female	EAE(MOG)	BMSC	Allogeneic	200μg	Kit	at 12, 15, 18 days	IV	28 days
8	Stem Cell-Derived Exosomes as Nanotherapeutics for Autoimmune and Neurodegenerative Disorders	Riazifar et al.(2019)USA (10)	mice	C57Bl/6	female	EAE(MOG)	BMSC	Xenogenic	150μg	Differential centrifugation	at day 18	IV	40 days
9	Exosomes derived from bone marrow mesenchymal stromal cells promote remyelination and reduce neuroinflammation in the demyelinating central nervous system	Zhang et al.(2020)USA (29)	mice	C57BL/6	female	EAE(MOG)	BMSC	Xenogenic	5 ×10^10 particles	Differential centrifugation	twice a week for 4 weeks initiated on day 10	IV	35 days
10	Therapeutic effects of extracellular vesicles from human adipose‐derived mesenchymal stem cells on chronic experimental autoimmune encephalomyelitis	Jafarinia et al.(2019)Iran (30)	mice	C57Bl/6	female	EAE(MOG)	ADMSC	Xenogenic	60 μg	Differential centrifugation	at day 10	IV	30 days
11	Human umbilical cord mesenchymal stem cell-derived extracellular vesicles attenuate experimental autoimmune encephalomyelitis *via* regulating pro and anti-inflammatory cytokines	Koohsari et al.(2021)Iran (31)	mice	C57Bl/6	female	EAE(MOG)	UCMSC	Xenogenic	50μg	Differential centrifugation	at day 9	IV	30 days
12	Intranasal administration of small extracellular vesicles derived from mesenchymal stem cells ameliorated the experimental autoimmune encephalomyelitis	Fathollahi et al.(2021)Iran (32)	mice	C57Bl/6	female	EAE(MOG)	ADMSC	Allogeneic	10 μg	Kit	at day 15th to 27th after immunization	IN	27 days

### Meta-analysis

**Figure 2:**
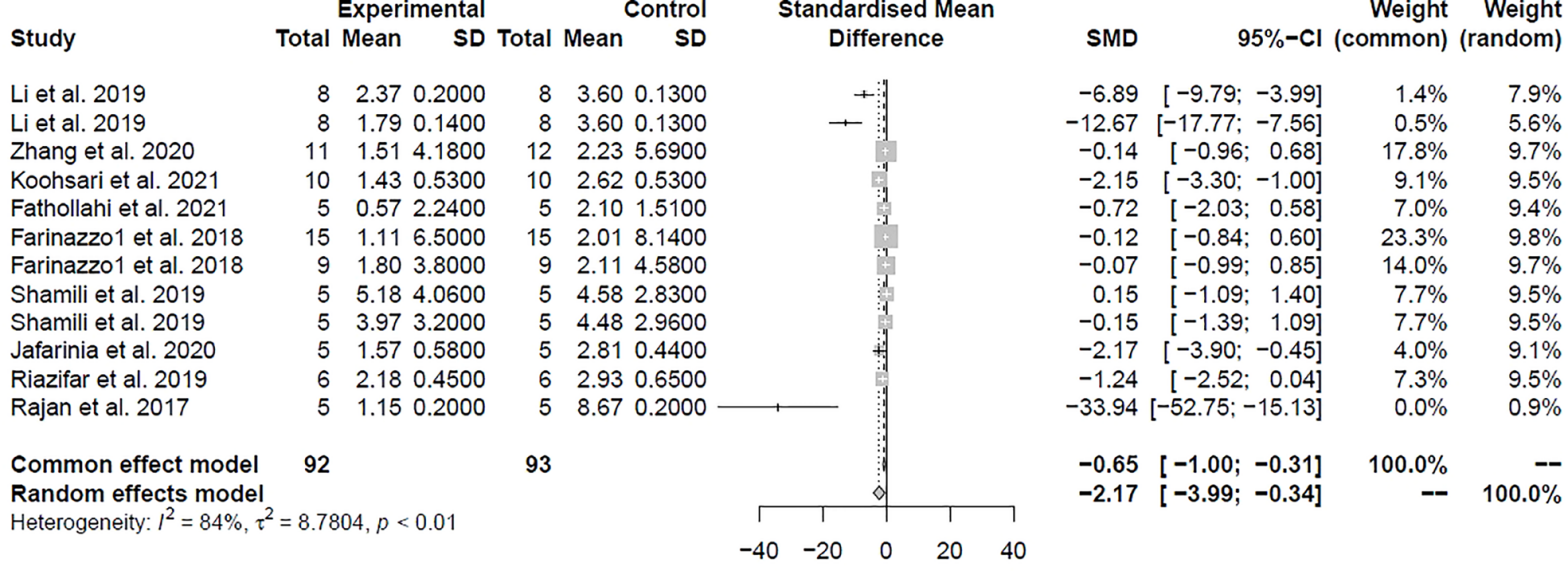
Forest plot shows the mean effect size and 95% confidence interval (CI) for clinical score.

A total of 12 comparisons of 11 studies involving 174 animals investigated the effect of MSC-EVs transplantation on MS in the EAE model by examining the clinical score in **Table 1**. On the basis of the clinical score, pooled analysis indicated that infusion of MSC-EVs did significantly improve the clinical symptoms of the EAE animals and delayed the progression of the disease compared to controls in both studies (SMD = -2.17, 95% CI: -3.99 to -0.34, P < 0.001). There was substantial heterogeneity among these studies (*I* ^2^ = 84%) (**Figure 2**).

### Exploration of heterogeneity and subgroup analysis

In our meta-analyses, pooled estimates exhibited considerable heterogeneity (I2 values exceeding 70% in all analyses). Therefore, we performed subgroup analyses. Stratified analyses were performed in order to further explore the impact of the source of heterogeneity and study design on the beneficial effect of MSC-EVs on EAE based on the type of MSCs, the source of the MSCs, the species and gender of the animal, the method by which EVs were extracted, the time at which the transplants were administered, and the route of exposure. The results of the stratified analyses are described in **Figures S1–8**. In regards to the clinical score, there was no significant difference in effect sizes associated with the route of admission (P = 0.17) (**Figure S1**), type of MSCs (P = 0.54) (**Figure S2**), time of administration (P = 0.11) (**Figure S3**), the method of extraction of MSC-EVs (P = 0.07) (**Figure S4**), number of times (P = 0.02) (**Figure S8**) and MSC-EVs dose (P = 0.21) (**Figure S9**). Nevertheless, significant differences in effect sizes and the source of heterogeneity were observed depending on animal species (P < 0.01) (**Figure S5**), the source of the MSCs (P < 0.01) (**Figure S6**), as well as the animal gender (P < 0.01) (**Figure S7**). When using MSC-EVs to treat MS, the effectiveness of the treatment is better in rats than in mice. In terms of stem cell utilization, the most effective of them are the PDLSCs, followed by BMSCs, UCMSCs, and ADSCs at the end. In animal models with male participants, MSC-EVs for males had a greater effect than female participants and not reported group.

### Sensitivity analysis

**Figure 3:**
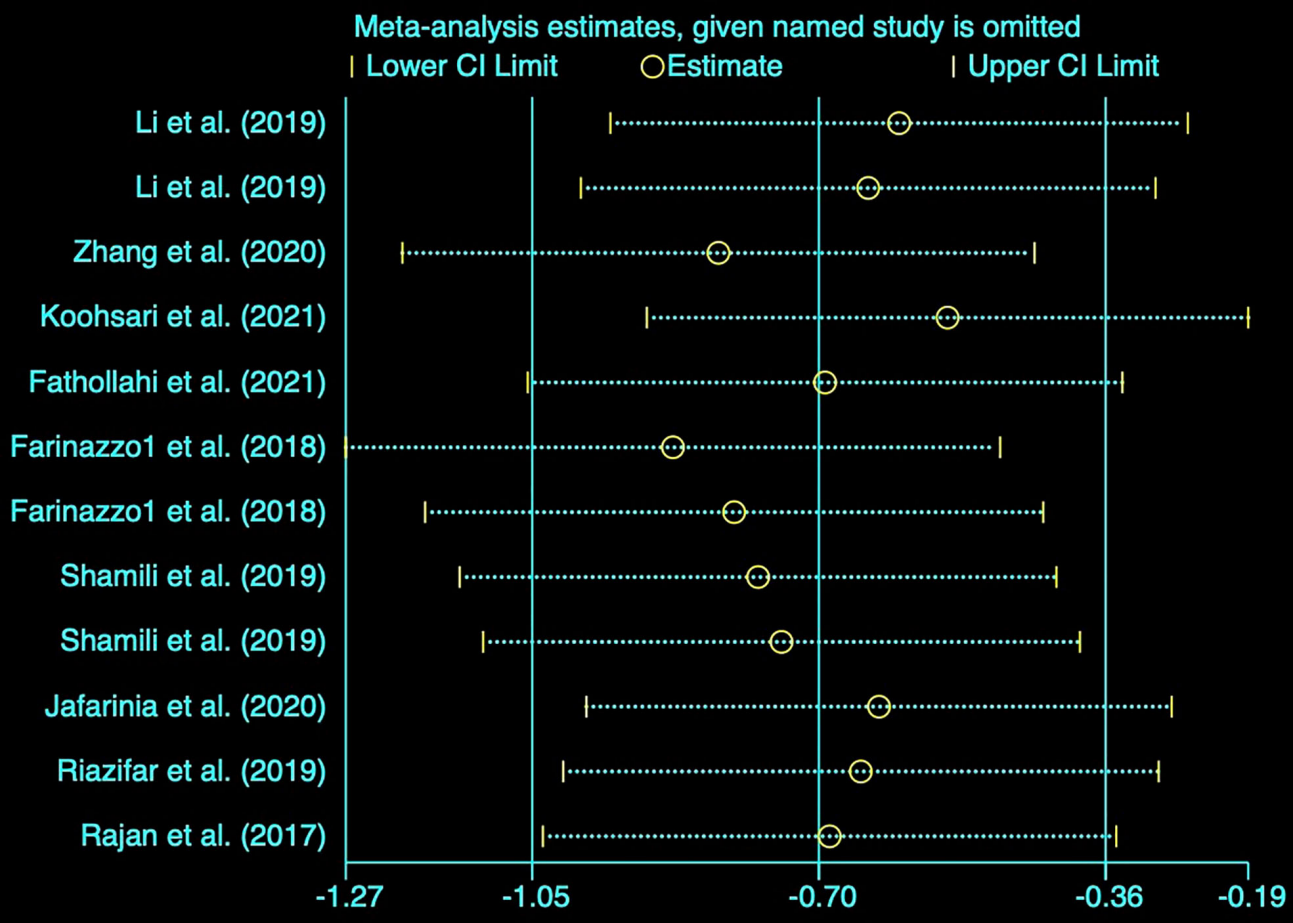
Sensitivity analysis of the studies included in clinical score.

Based on the notable heterogeneity, we performed a sensitivity analysis to determine the stability of results by sequentially excluding each research study. The clinical score pooled SMD as shown in **Figure 3** was not affected by any of the studies.

### Publication bias

**Figure 4:**
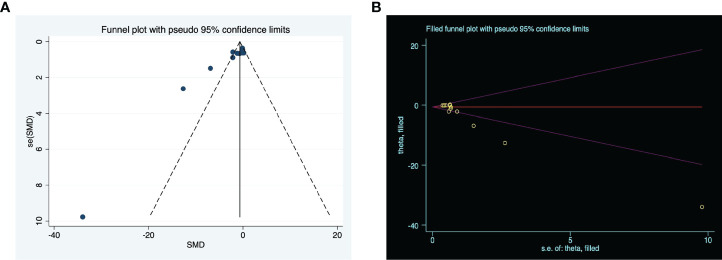
The evaluation of publication bias. **(A)** Funnel plots for clinical score, with the y-axis signifying study quality and the x-axis showing the study results. **(B)** Trim-and-fill method was used to evaluate the missing studies in clinical score. SMD, standardized mean difference.

Generally, studies with statistically significant effects have a higher rate of publication than studies with a negative outcome. Upon visual inspection, the funnel plots for the meta-analysis appeared asymmetrical (P = 0.000) (**Figure 4A**) suggesting possible publication bias among the included studies. Consequently, we applied the trim-and-fill methodology to evaluate missing studies and recalculated the pooled effect. This analysis suggests that the impact of clinical score is similar to the previous findings (SMD: -1.461, 95% confidence interval: -2.431 to -0.491, P = 0.003), suggesting that no missing studies are available (**Figure 4B**).

### Assessment of RoB

**Table 2** summarizes the risk of bias across included studies. No studies were judged to have a low risk of bias across all domains. Selection bias was unclear in most studies when examining randomization, and few studies described baseline characteristics of included animals. In addition, no studies specifically described the method by which random sequences were generated. For all the studies, allocation concealment, random housing, random outcome, and other aspects of bias assessment were not mentioned. Approximately 30% (n = 4/12) of the included studies mentioned blinding during outcome assessment. Attrition bias was variable across studies, with a high risk of bias assigned to seven studies that did not account for the decrease in animal numbers reported between methods and results. Three studies had a low risk of attrition bias, and the remainder were unclear. Selective reporting bias was unclear across all studies, as published protocols were not available. We did not identify any other sources of bias.

**Table 2:** SYRCLE risk of bias assessment for included studies.

Author year Country	selection bias			performance bias		detection bias		attrition bias	reporting bias	other bias
	Random sequence generation?	Groups similar at baseline?	Allocation concealed?	Animals randomly housed?	Blinding of caregivers and/or examiners?	Random selection for outcome assessment?	Blinding of outcome assessor?	Incomplete outcome data addressed?	Free from selective outcome reporting?	Free from other bias?
**Rajan et al.(2017)Italy (** 25)	**U**	**U**	**U**	**U**	**U**	**U**	**U**	**L**	**U**	**U**
**Farinazzo1 et al.(2018)Italy (** 26)	**U**	**U**	**U**	**U**	**U**	**U**	**L**	**U**	**U**	**U**
**Farinazzo1 et al.(2018)Italy (** 26)	**U**	**U**	**U**	**U**	**U**	**U**	**L**	**U**	**U**	**U**
**Li et al.(2019)A China (** 27)	**U**	**U**	**U**	**U**	**L**	**U**	**L**	**L**	**U**	**U**
**Li et al.(2019)B China (** 27)	**U**	**U**	**U**	**U**	**L**	**U**	**L**	**L**	**U**	**U**
**Shamili et al.(2019)Iran (** 28)	**U**	**U**	**U**	**U**	**U**	**U**	**U**	**H**	**U**	**U**
**Shamili et al.(2019)Iran (** 28)	**U**	**U**	**U**	**U**	**U**	**U**	**U**	**H**	**U**	**U**
**Riazifar et al.(2019)USA (** 10)	**U**	**U**	**U**	**U**	**U**	**U**	**U**	**H**	**U**	**U**
**Zhang et al.(2020)USA (** 29)	**U**	**U**	**U**	**U**	**U**	**U**	**U**	**H**	**U**	**U**
**Jafarinia et al.(2019)Iran (** 30)	**U**	**U**	**U**	**U**	**U**	**U**	**U**	**H**	**U**	**U**
**Koohsari et al.(2021)Iran (** 31)	**U**	**U**	**U**	**U**	**U**	**U**	**U**	**H**	**U**	**U**
**Fathollahi et al.(2021)Iran (** 32)	**U**	**U**	**U**	**U**	**U**	**U**	**U**	**H**	**U**	**U**

## Discussion

### Main findings

The therapeutic potential of MSCs has been increasingly studied over the past decade for various CNS diseases (10). MSC-based cell therapy is one method of treating MS (33, 34). Currently, there are more than 9 MSCs clinical trials underway or completed to investigate these diseases (https://clinicaltrials.gov/ct2/home) (**Table 3**). With the advancement of research, growth in recognition of and praise for the paracrine function of MSCs has increased. As the most significant part of paracrine, EVs have become a new research hotspot and are even being tested in clinical studies (35). In this study, our results showed that rodents with MS benefited from MSC-EVs therapy as manifested by significant amelioration of functional outcomes, providing insight into the potential therapeutic applications of MSC-EVs in preclinical studies of MS. The use of MSC-EVs therapy in MS has not been implemented in the treatment management of patients with MS despite preclinical studies showing that MSC-EVs could improve MS. Since animal experiments serve as a basis for designing clinical trials, it is imperative to examine the combined effects of preclinical and clinical studies. For MSC-EVs, several issues remain unclear concerning delivery timing, routes of administration, and dosage, especially in the current situation where there is no unified transplant unit.

**Table 3:** Clinical studies using MSCs for treatment of MS.

Number	No. NCT	Years	Study Title	Locations	Recruitment status	Phase	Ages (years)	Allo/Auto	Route of administration	Stem Cell Source	No. of SCs	Follow-up Period
1	NCT02166021	2015	Clinical Efficacy of Autologous Mesenchymal Bone Marrow Stem Cells in Active and Progressive Multiple Sclerosis	Israel	Completed	Phase 2	18 Years to 65 Years	Allogeneic	intrathecally (IT) or intravenously (IV)	bone marrow	15 × 10^6^/mL.	12 months
2	NCT00781872	2006	Mesenchymal Stem Cells for the Treatment of MS	Israel	Completed	Phase 1 Phase 2	35 Years to 65 Years (Adult, Older Adult)	Allogeneic	intrathecally (IT) or intravenously (IV)	bone marrow	6 × 10^6^ cells intrathecally and 2 × 10^6^ cells intravenously	1-4 year
3	NCT03799718	2019	Safety and Efficacy of Repeated Administration of NurOwn (MSC-NTF Cells) in Participants With Progressive MS	USA	Completed	Phase 2	18 Years to 65 Years	Autologous	intrathecally (IT)	Mesenchymal Stem Cells Secreting Neurotrophic Factors	not reported	12 weeks
4	NCT02239393	2015	Safety and Efficacy of Intravenous Autologous Mesenchymal Stem Cells for MS: a Phase 2 Proof of Concept Study	Canada	Completed	Phase 2	18 Years to 50 Years (Adult)	Autologous	intravenously (IV)	not reported	1~2 × 10^6^ cells/kg	24 weeks
5	NCT00813969	2008	Autologous Mesenchymal Stem Cell (MSC) Transplantation in MS	USA	Completed	Phase 1	18 Years to 55 Years (Adult)	Autologous	intravenously (IV)	not reported	2 × 10^6^ cells per kg	6 months
6	NCT04823000	2021	Effects of Repeated Mesenchymal Stem Cells (MSC) in Patients With Progressive Multiple Sclerosis	Israel	Completed	Phase 1 Phase 2	18 Years to 65 Years (Adult, Older Adult)	Autologous	intrathecally (IT) or intravenously (IV)	not reported	1 × 10^6^ per kg of body weight	12 months
7	NCT03326505	2017	Allogenic Mesenchymal Stem Cells And Physical Therapy for MS Treatment	Jordan	Completed	Phase 1 Phase 2	18 Years to 65 Years (Adult, Older Adult)	Allogeneic	intrathecally (IT)	Umbilical cord derived Mesenchymal Stem Cells	1 × 10^6^ cells	12 months
8	NCT05116540	2021	Randomized Double-Blind Phase 2 Efficacy and Safety of Autologous HB-MSCs vs Placebo for Treatment of Multiple Sclerosis	USA	Recruiting	Phase 2	18 Years to 75 Years (Adult, Older Adult)	Autologous	intravenously (IV)	Adipose derived Mesenchymal stem cells (Autologous)	not reported	52 weeks
9	NCT01228266	2010	Mesenchymal Stem Cell Transplantation in MS	Spain	Terminated	Phase 2	18 Years to 50 Years (Adult)	Autologous	not reported	not reported	2 × 10^6^ cells per Kg	12 months

As a result of our subgroup analysis, we found that in the EV-treated MS model, the treatment effect was superior in male mice than in female mice. There may be a correlation between this finding and gender characteristics of MS. The incidence of MS is higher in women than in men, and women are generally twice as common as men, as well as having a more severe condition (36). The treatment effect of the treatment was more evident in male mice than in female mice, but the results were less robust because of the small sample size. Furthermore, results revealed that rats possess greater therapeutic effects than mice. However, it is difficult to establish stable rat models of EAE, even though several studies have used Lewis Rats (37), DA Rats (38), and SD Rats (39) to induce the EAE model; mice are primarily used in these studies. Since there is limited information regarding rats, systematic comparative studies will be required to verify this conjecture in the future. Additionally, we have found that PDLSCs have a better therapeutic effect than other types of MSCs, followed by BMSCs, UCMSCs, and ADSCs. In contrast to PDLSCs, which derive from ectoderm, BMSCs, UCMSCs, and ADSCs draw their origins from mesoderm, while MS is a disorder of the nervous system. Thus, it is worth considering whether the differences in the effects of such treatments can be attributed to differences in the germ layers from which they are formed. In the study by Payne et al., the researchers reported that BM-MSCs, ASCs, and UC-MSCs did not possess any beneficial effects on animal models of MS when MSCs were administered after the onset of symptoms (40). Since there is a limited amount of information on MSCs, more systematic comparative studies will be required in the future to verify this conjecture. Previous research has demonstrated that MSCs have beneficial effects regardless of their source (41). As noted by Zhu et al. (42), the administration of syngeneic and allogeneic MSCs did not influence the improvement of EAE clinical symptoms in animal models of MS, which is consistent with our findings. Based on our results, there were no statistically significant differences between doses, which may be due to the lack of literature included in our study. This comparison needs to be verified by more investigations. As part of the determination of the role of MSC-EVs, it is also necessary to consider the timing and number of injections. However, MSC-EVs are capable of promoting functional recovery regardless of the number and duration of injections. These results agree with those reported by Bai et al. (19), and Donders et al. (43) in their article on MSC therapy for MS, that no matter the injection time, MSCs can promote functional recovery (19, 43). Generally, the various subgroup analyses above can only produce hypotheses, not confirm them. The results of the subgroup analysis are speculative since they are based on a reanalysis of published data and not a well-created randomized controlled trial. Therefore, even though the subgroup analysis provided updated evidence, these findings should be interpreted with caution.

By exploring the therapeutic effect of MSC-EVs in MS, the application strategy of MSC-EVs will be improved, in turn resulting in the clinical application of MSC-EVs (44, 45). Despite numerous preclinical studies showing the potential of MSC-EVs in regenerative medicine, no detailed studies have yet been conducted to examine the mechanisms involved in its recovery. Based on current preclinical studies, it appears that SC-EVs have the potential to treat MS models associated with anti-inflammatory, pro-myelinating, immunomodulatory, and neuroprotective properties. (a) anti-inflammatory. MSC-EVs may exhibit anti-inflammatory effects not only by suppressing the infiltration of leukocytes but also by reducing the release of pro-inflammatory factors such as s IL-17, IFN-γ, IL-1β, IL-6, and TNF-α (46). Rajan et al. noted that EVs obtained from PDLSCs modulate NF-κB levels, and inhibit NALP3 inflammasome activation (25). In the other study, injection of MSC-EVs infusion resulted in a reduction in neuroinflammation both through inhibiting the activation of microglia as well as promoting a shift of pro-inflammatory microglia (M1) into anti-inflammatory microglia (M2) (27) (b) Remyelination effect. Studies have demonstrated that MSC-EVs can promote the proliferation of oligodendrocytes and induce remyelination. (c) Immunomodulatory. Our current understanding of MS pathogenesis is concerned with the critical role of the immune system in disease onset. Many reviews have reported that CD4+ T cells play a crucial role in the pathogenesis of MS. Additionally, regulatory T cells (Tregs) are critical players in the pathogenesis of MS autoimmune inflammation (47). Previous studies showed that intravenous administration of ADSC-EVs attenuates induced-EAE through diminishing proliferative potency of CD4+T cells (30). In another study, Riazifar et al. demonstrated that MSC-EVs upregulated the number of CD4+CD25+FOXP3+ regulatory T cells (Tregs) within the spinal cords of EAE mice (10). (d) Neuroprotection. According to Rajan et al., MSC-EVs reduced the expression of apoptosis markers STAT1, p53, cleaved caspase 3, and Bax in EAE mice, which partially contribute to neuroprotection.

### Clinical challenges of MSC-EVs therapy for MS

**Figure 5:**
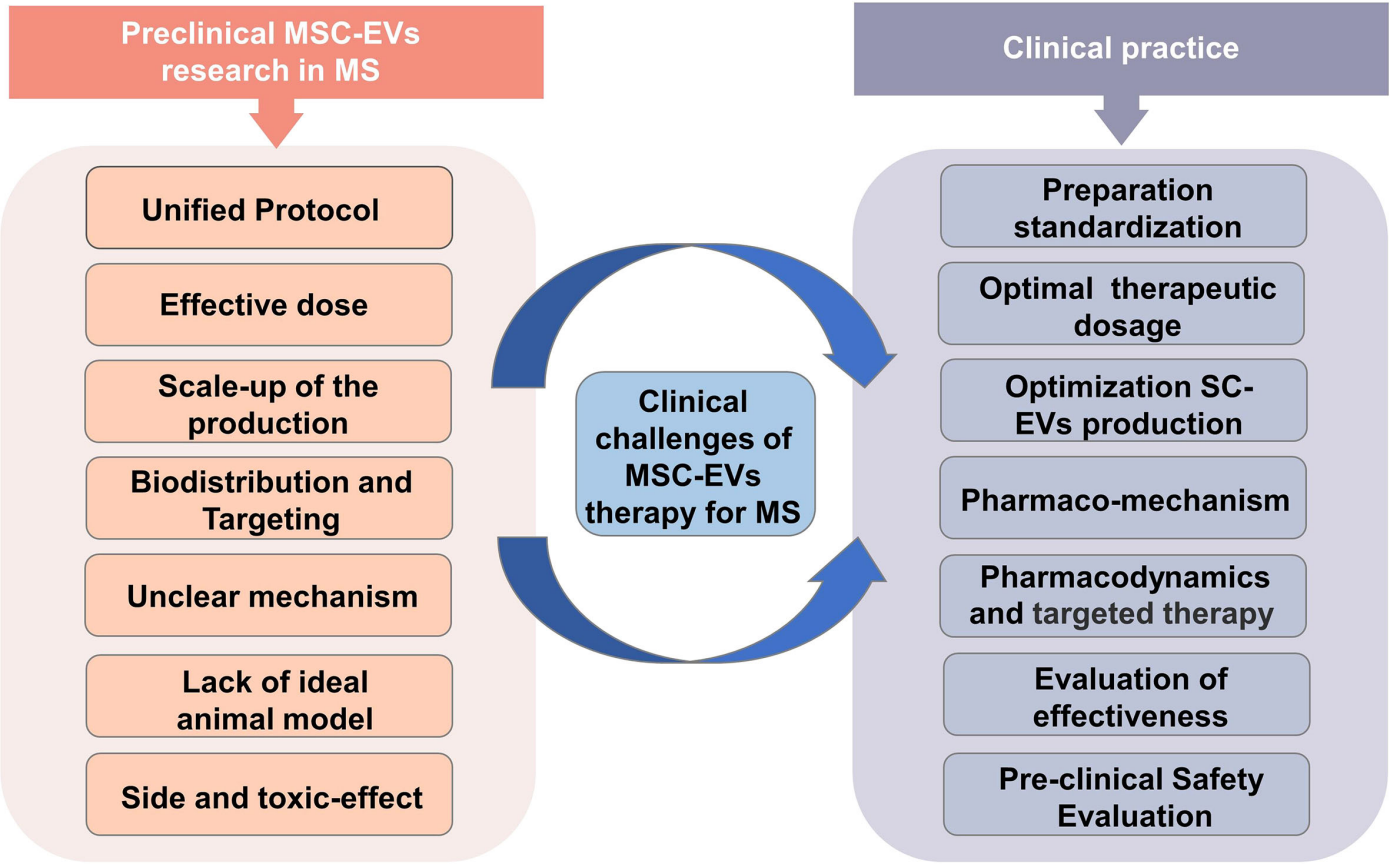
Clinical challenges of MSC-EVs therapy for MS.

While MSC-EVs may have therapeutic potential for patients with MS, there are several challenges associated with their use (**Figure 5**). (a) The extraction method of MSC-EVs. Initial consideration should be given to the technical challenge, which encompasses everything from isolating the EV to characterization and standardization of it for clinical use. Low-throughput techniques like ultrasound centrifugation are usually used to isolate MSC-EVs in most studies. This calls for advanced methods and techniques. (b) The scaling up of the MSC culture. The scaling up of MSC culture is another substantial technical challenge that must be addressed to produce adequate quantities of MSC-EVs for clinical trials. Bioreactors and 3D stem cell culture may be able to solve this problem (48, 49). However, even though bioreactors are optimal culture media, they may induce cellular stress upon the cells, and therefore EVs may be less effective if this stress occurs (50–52). Hence further research is required to develop suitable methods for large-scale cultivation. (c) Effective dose. The determination of the appropriate dosage and mode of action for therapeutic MSC-EVs remains a challenging task in this field. MSC-EVs were dosed in different ways in various studies, including absolute protein amounts, particle numbers, the amount of EVs released by a specific number of MSCs, or EVs released continuously or dosed by body weight. It is therefore essential to establish a standardized dosage unit in order to determine the optimal therapeutic dose. (d) Biodistribution and Targeting of MSC-EV to Target Tissues. To investigate MSC-EV as a therapeutic tool, it is critical to consider their biodistribution and targeting mechanisms *in vivo*. In a mouse model of EAE, Riazifar et al. (2019) (10) labeled MSC-EV using DiR, which is a lipophilic dye commonly used for *in vivo* and ex vivo imaging. After the MSC-EVs injection of 3 h, freshly dissected tissues of mice were analyzed immediately for the fluorescence signal using an IVIS imaging system. They found that most EVs were found in the liver and spleen of healthy and EAE mice. Furthermore, the absence of signal in the lungs of MSC-EV-treated animals suggests that EVs, unlike MSCs, bypass the small lung vasculature bed due to their small size. Interestingly, dye-labeled EVs were detected in the spinal cords of EAE animals, but not in healthy animals, at 3 hours following administration, indicating that MSC-EVs are involved in causing the lesions. Another study from Zhang et al. (2022) (29) used CD63-GFP to label MSC-EVs. They found that MSC-EVs crossed the BBB four hours after IV administration by laser scanning confocal microscopy of immunofluorescent staining, with green GFP signals present in the CNS and internalized by parenchymal cells. Additionally, MSC-EVs colocalized with Oligodendrocyte Progenitor Cells (OPCs) using double immunofluorescence staining combined with PDGFRa (OPC marker). Considering that the number of relevant studies and the MSC-EVs targeting of articles are still inadequate, other strategies for improving the targeting of SC-EVs may also be considered. (e) Unclear mechanism. Identifying the mechanisms of action of therapeutics containing MSC-EVs is another challenge in this area. Developing dose and functional assessments will be easier with a deeper understanding of MSC-EVs mechanism of action. Therefore, once we can gain a full understanding of the therapeutic potential of MSC-EVs, we may be able to optimize their extraction to achieve higher levels of function. (f) Lack of MS model. EAE is not the only MS model that causes demyelination and inflammation (53). Other models of MS are caused by viruses (54) and toxins like cuprizone (55) and ethidium bromide (56). In general, these models exhibit distinct histopathological differences. Studies have shown that MS can be successfully modeled using EAE and viral-induced demyelination/inflammation models. However, toxins-induced demyelination models are more suitable for simulating specific mechanisms of myelin regeneration and degeneration (57). Although the EAE model is one of the most commonly recognized models of MS, since EAE is not equivalent to MS, further research is necessary to establish the efficacy of MSC-EV in treating MS in preclinical trials. (g) Effects on safety and toxicity. With any treatment, it is necessary to establish a safety profile. Research on the safety and side effects of MSC-EV remains insufficient. Regarding SCs therapy, the main concerns are tumorigenicity, immunogenicity, and genomic mutability (58–60). Fortunately, MSC-EVs do not suffer from the limitations described above. Although there are still technical and regulatory challenges, progressively more studies show that MSC-EVs have substantial potential for therapeutic use.

### Limitations

To our knowledge, this is the first systematic review of animal studies assessing the therapeutic efficacy of MSC-EVs in the treatment of MS rodent models. However, there are several limitations to this meta-analysis. In the first place, the number of studies currently available that can be included in the meta-analysis was small. For a more comprehensive investigation of these issues, larger and well-designed preclinical studies are necessary. Secondly, though we performed subgroup analyses and sensitivity analyses, this does not significantly reduce the heterogeneity between studies, which may weaken the stability of the results. Thirdly, we included stem cell-derived EVs as well as other cell-derived EVs, although we did not make a direct comparison between them to identify the most suitable option, which may have also contributed to the higher degree of heterogeneity. Fourthly, there was a possibility that the research studies identified might not be completely retrieved, which may have created publication bias. Last but not least, data extraction from graphics using GetData Graph Digitizer software may have altered the original data, which may have also affected the results.

## Conclusions

Overall, our systematic review and meta-analysis provide a comprehensive assessment of the available evidence on the efficacy of MSC-EVs therapy for MS in preclinical rodent models, suggesting that MSC-EV therapy exhibits prospective beneficial effects in MS. Future preclinical studies should be designed with a strong focus on methodological rigor. Well-designed studies will contribute to a better understanding of the benefits of MSC-EVs therapy for MS.

## Data availability statement

The original contributions presented in the study are included in the article/**Supplementary Material**. Further inquiries can be directed to the corresponding authors.

## Author contributions

ZH supervised the project. LG, JZ, and HD analyzed the data. CX extracted the data. LG and ZH wrote the paper. All authors contributed to the article and approved the submitted version.

## Funding

This work was supported by National Natural Science Foundation of China (No.81974213), Key Project of Hunan Provincial Science and Technology Innovation (2020SK2102), Hunan Provincial Natural Science Foundation of China (2021JJ40830).

## Conflict of interest

The authors declare that the research was conducted in the absence of any commercial or financial relationships that could be construed as a potential conflict of interest.

## Publisher’s note

All claims expressed in this article are solely those of the authors and do not necessarily represent those of their affiliated organizations, or those of the publisher, the editors and the reviewers. Any product that may be evaluated in this article, or claim that may be made by its manufacturer, is not guaranteed or endorsed by the publisher.
