# Supplementary material for: Mesenchymal stromal cell extracellular vesicles for multiple sclerosis in preclinical rodent models: A meta-analysis
Source: Front Immunol. 2022 Nov 4;13:972247. doi: 10.3389/fimmu.2022.972247 (PMC9673165; doi:10.3389/fimmu.2022.972247)
Supplement: Supplementary file 1 [file DataSheet_1.doc]

**Supplement [Materials](../../../../D:/%25E8%25BD%25AF%25E4%25BB%25B6/360%25E6%25B8%25B8%25E8%25A7%2588%25E5%2599%25A8/Youdao/Dict/8.9.9.0/resultui/html/index.html#/javascript:;)**

**Additional file 1**

**The Detailed Search Strategy**

**Pubmed：**

((((((Sclerosis, Multiple[Title/Abstract]) OR (Sclerosis, Disseminated[Title/Abstract])) OR (Disseminated Sclerosis[Title/Abstract])) OR (MS[Title/Abstract])) OR ("Multiple Sclerosis"[Mesh])) OR ((((((((((((("Allergic Encephalomyelitis, Experimental"[Title/Abstract]) OR ("Encephalomyelitis, Experimental Allergic"[Title/Abstract])) OR ("Experimental Allergic Encephalomyelitides"[Title/Abstract])) OR ("Autoimmune Encephalomyelitis, Experimental"[Title/Abstract])) OR ("Experimental Autoimmune Encephalomyelitis"[Title/Abstract])) OR ("Encephalomyelitis, Allergic"[Title/Abstract])) OR ("Encephalomyelitis, Experimental Autoimmune"[Title/Abstract])) OR ("Experimental Allergic Encephalomyelitis"[Title/Abstract])) OR ("Allergic Encephalomyelitis"[Title/Abstract])) OR ("Autoimmune Experimental Encephalomyelitis"[Title/Abstract])) OR ("Encephalomyelitis, Autoimmune Experimental"[Title/Abstract])) OR ("Experimental Encephalomyelitis, Autoimmune"[Title/Abstract])) OR ("Encephalomyelitis, Autoimmune, Experimental"[Mesh])))

**AND**

((((Extracellular Vesicle[Title/Abstract]) OR (Vesicle, Extracellular[Title/Abstract]) OR (Vesicles, Extracellular[Title/Abstract]) OR (Exovesicles[Title/Abstract]) OR (Exovesicle[Title/Abstract]) OR (Apoptotic Bodies[Title/Abstract]) OR (Apoptotic Body[Title/Abstract]) OR (Bodies, Apoptotic[Title/Abstract]) OR (Body, Apoptotic[Title/Abstract]) OR (Extracellular Vesicles[MeSH Major Topic])) OR (Exosomes[MeSH Major Topic]) OR (Endosome[Title/Abstract]) OR (Receptosomes[Title/Abstract]) OR (Receptosome[Title/Abstract]))) OR ((Endosomes[MeSH Major Topic]) OR (Cell Derived Microparticles[Title/Abstract]) OR (Cell Derived Microparticle[Title/Abstract]) OR (Microparticle, Cell Derived[Title/Abstract]) OR (Ectosomes[Title/Abstract]) OR (Ectosome[Title/Abstract]) OR (Microparticles, Cell Derived[Title/Abstract]) OR (Microparticles, Cell Derived[Title/Abstract]) OR (Shedding Microvesicles[Title/Abstract]) OR (Microvesicle, Shedding[Title/Abstract]) OR (Microvesicles, Shedding[Title/Abstract]) OR (Shedding Microvesicle[Title/Abstract]) OR (Cell Membrane Microparticles[Title/Abstract]) OR (Cell Membrane Microparticle[Title/Abstract]) OR (Membrane Microparticle, Cell[Title/Abstract]) OR (Membrane Microparticles, Cell[Title/Abstract]) OR (Microparticle, Cell Membrane[Title/Abstract]) OR (Microparticles, Cell Membrane[Title/Abstract]) OR (Circulating Cell Derived Microparticles[Title/Abstract]) OR (Cell Derived Microparticle, Circulating[Title/Abstract]) OR (Cell Derived Microparticles, Circulating[Title/Abstract]) OR (Circulating Cell Derived Microparticles[Title/Abstract]) OR (Circulating Cell Derived Microparticle[Title/Abstract]) OR (Microparticle, Circulating Cell Derived[Title/Abstract]) OR (Microparticles, Circulating Cell Derived[Title/Abstract]) OR (Cell Derived Microparticles[MeSH Major Topic])))

**Web of science**

**#1**

TS=(“Sclerosis, Multiple” OR “Sclerosis, Disseminated” OR “Disseminated Sclerosis” OR “MS” OR “multiple sclerosis”)

**#2**

**TS=(“Allergic Encephalomyelitis, Experimental” OR “Encephalomyelitis, Experimental Allergic” OR “Experimental Allergic Encephalomyelitides” OR “Autoimmune Encephalomyelitis, Experimental” OR “Experimental Autoimmune Encephalomyelitis”OR “Encephalomyelitis, Allergic” OR “Encephalomyelitis, Experimental Autoimmune” OR “Experimental Allergic Encephalomyelitis” OR “Allergic Encephalomyelitis” OR “Autoimmune Experimental Encephalomyelitis” OR “Encephalomyelitis, Autoimmune Experimental” OR “Experimental Encephalomyelitis, Autoimmune”)**

**#3=#1 OR #2**

**#4**

TS=(“Exosomes” OR “Endosome” OR “Receptosomes” OR “Receptosome” OR “Endosomes” OR “Cell Derived Microparticles” OR “Cell-Derived Microparticle” OR “Microparticle, Cell-Derived” OR “Ectosomes” OR “Ectosome” OR “Microparticles, Cell-Derived” OR “Microparticles, Cell Derived” OR “Shedding Microvesicles” OR “Microvesicle, Shedding” OR “Microvesicles, Shedding” OR “Shedding Microvesicle” OR “Cell Membrane Microparticles” OR “Cell Membrane Microparticle” OR “Membrane Microparticle, Cell” OR “Membrane Microparticles, Cell” OR “Microparticle, Cell Membrane” OR “Microparticles, Cell Membrane” OR “Circulating Cell-Derived Microparticles” OR “Cell-Derived Microparticle, Circulating” OR “Cell-Derived Microparticles, Circulating” OR “Circulating Cell Derived Microparticles” OR “Circulating Cell-Derived Microparticle” OR “Microparticle, Circulating Cell-Derived” OR “Microparticles, Circulating Cell-Derived” OR “Cell-Derived Microparticles”)

**#5**

TS=(“Extracellular Vesicle” OR “Vesicle, Extracellular” OR “Vesicles, Extracellular” OR “Exovesicles” OR “Exovesicle” OR “Apoptotic Bodies” OR “Apoptotic Body” OR “Bodies, Apoptotic” OR “Body, Apoptotic” OR “Extracellular Vesicles”)

**#6=#4 OR #5**

**#7=#3 AND #6**

**Embase**

**#1**

('experimental autoimmune encephalomyelitis'/exp) OR ('experimental encephalomyelitis, autoimmune':ab,ti) OR ( 'encephalomyelitis, autoimmune experimental':ab,ti) OR ('autoimmune experimental encephalomyelitis':ab,ti) OR ('allergic encephalomyelitis':ab,ti)

OR ('experimental allergic encephalomyelitis':ab,ti) OR ('encephalomyelitis, experimental autoimmune':ab,ti) OR ('encephalomyelitis, allergic':ab,ti) OR ('experimental autoimmune encephalomyelitis':ab,ti) OR ('autoimmune encephalomyelitis, experimental':ab,ti) OR ('experimental allergic encephalomyelitides':ab,ti) OR ('encephalomyelitis, experimental allergic':ab,ti) OR ('allergic encephalomyelitis, experimental':ab,ti)

**#2**

'multiple sclerosis'/exp

**#3**

'sclerosis, multiple':ti,ab

**#4**

'sclerosis, disseminated':ti,ab

**#5**

'disseminated sclerosis':ti,ab

**#6**

'ms':ti,ab

**#7= #2 OR #3 OR #4 OR #5 OR#6**

**#8=#1OR #7**

**#9**

'extracellular vesicle':ab,ti OR 'vesicle, extracellular':ab,ti OR 'vesicles, extracellular':ab,ti OR 'exovesicles':ab,ti OR 'exovesicle':ab,ti OR 'apoptotic bodies':ab,ti OR 'apoptotic body':ab,ti OR 'bodies, apoptotic':ab,ti OR 'body, apoptotic':ab,ti OR 'extracellular vesicles'/exp OR 'exosomes'/exp OR 'endosome':ab,ti OR 'receptosomes':ab,ti OR 'receptosome':ab,ti OR 'endosomes'/exp OR 'cell derived microparticles':ab,ti OR 'cell-derived microparticle':ab,ti OR 'microparticle, cell-derived':ab,ti OR 'ectosomes':ab,ti OR 'ectosome':ab,ti OR 'microparticles, cell-derived':ab,ti OR 'microparticles, cell derived':ab,ti OR 'shedding microvesicles':ab,ti OR 'microvesicle, shedding':ab,ti OR 'microvesicles, shedding':ab,ti OR 'shedding microvesicle':ab,ti OR 'cell membrane microparticles':ab,ti OR 'cell membrane microparticle':ab,ti OR 'membrane microparticle, cell':ab,ti OR 'membrane microparticles, cell':ab,ti OR 'microparticle, cell membrane':ab,ti OR 'microparticles, cell membrane':ab,ti OR 'circulating cell-derived microparticles':ab,ti OR 'cell-derived microparticle, circulating':ab,ti OR 'cell-derived microparticles, circulating':ab,ti OR 'circulating cell derived microparticles':ab,ti OR 'circulating cell-derived microparticle':ab,ti OR 'microparticle, circulating cell-derived':ab,ti OR 'microparticles, circulating cell-derived':ab,ti OR 'cell-derived microparticles'/exp

**#10=#8 AND #9**

**Fig. S1. Subgroup analysis by delivery route for clinical score (intravenous versus intranasal administration ) .**

**
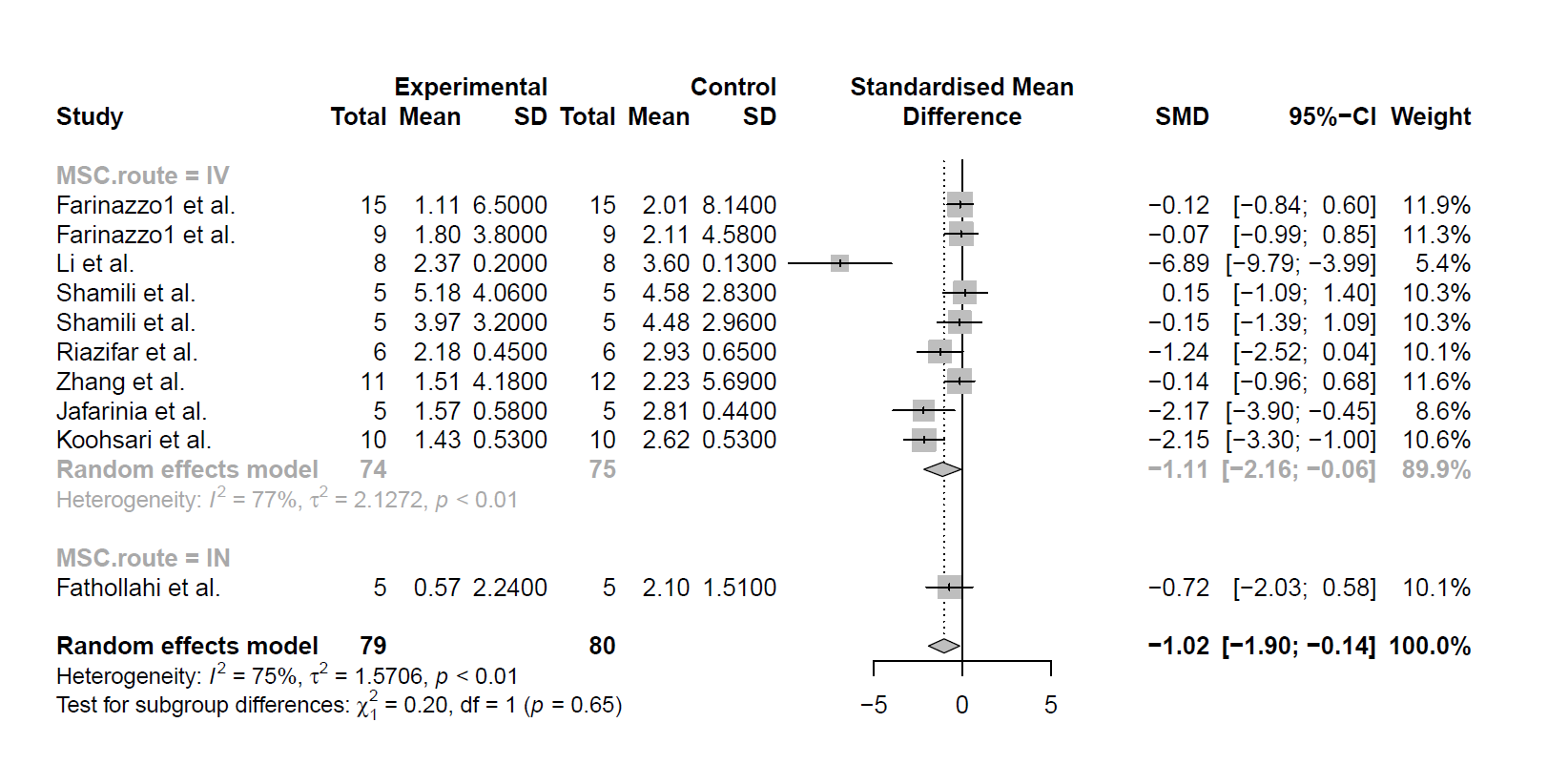
**

**Fig. S2. Subgroup analysis by the type of MSCs for the clinical score.**

**
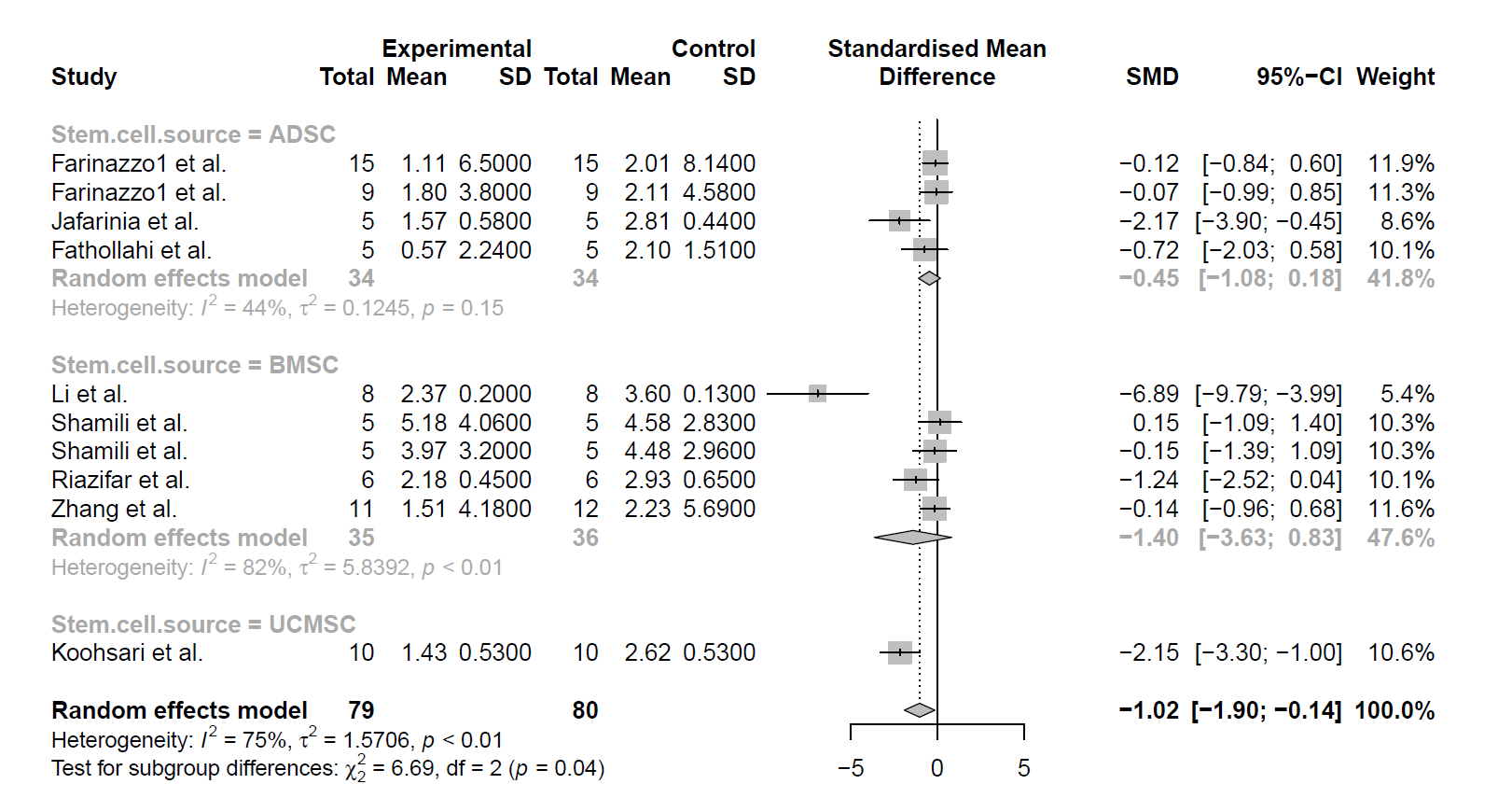
**

**Fig. S3. Subgroup analysis by the administration time for the clinical score.**

**
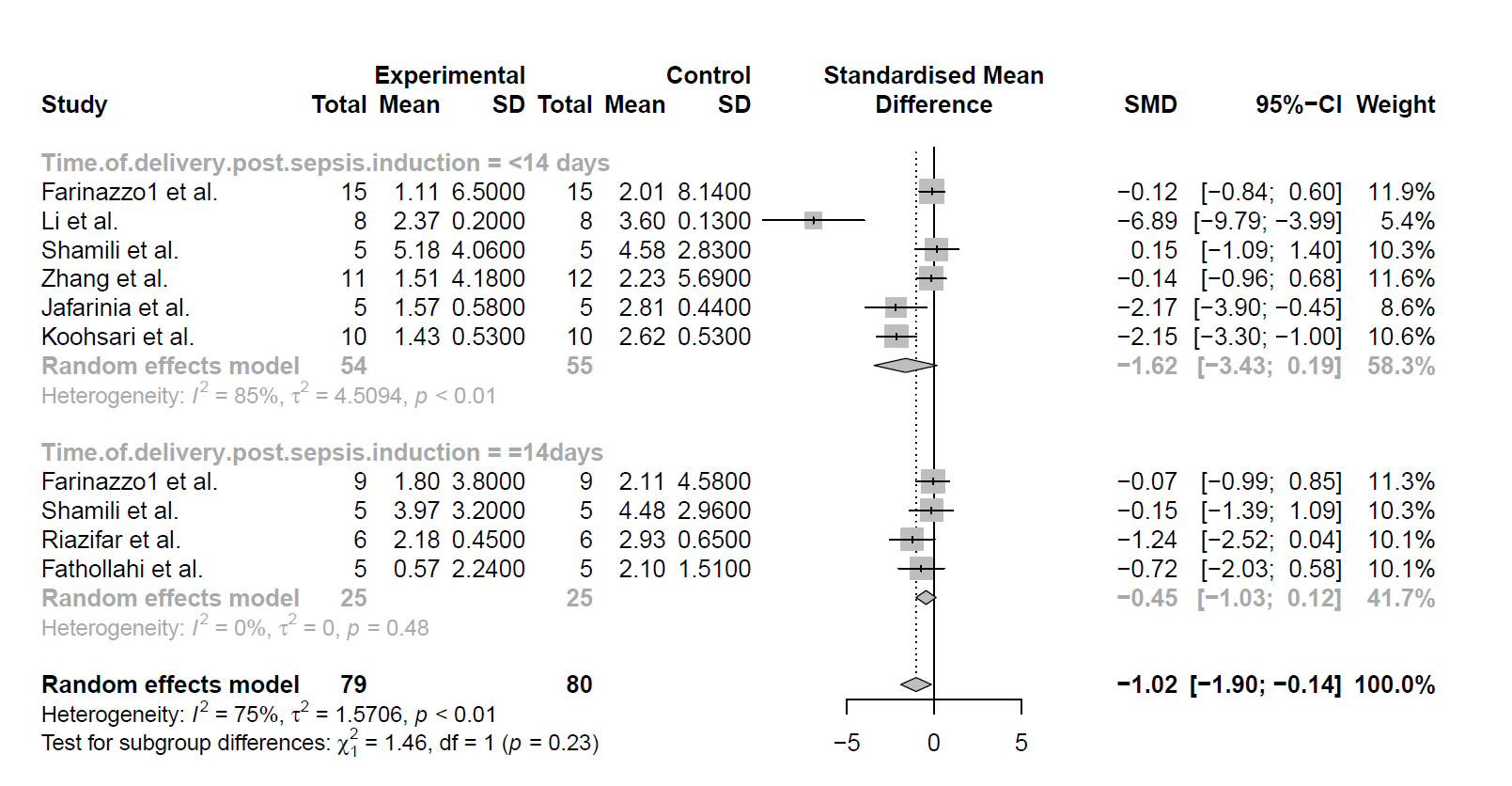
**

**Fig. S4. Subgroup analysis by the the method of extraction of MSC-EVs for the clinical score.**

**
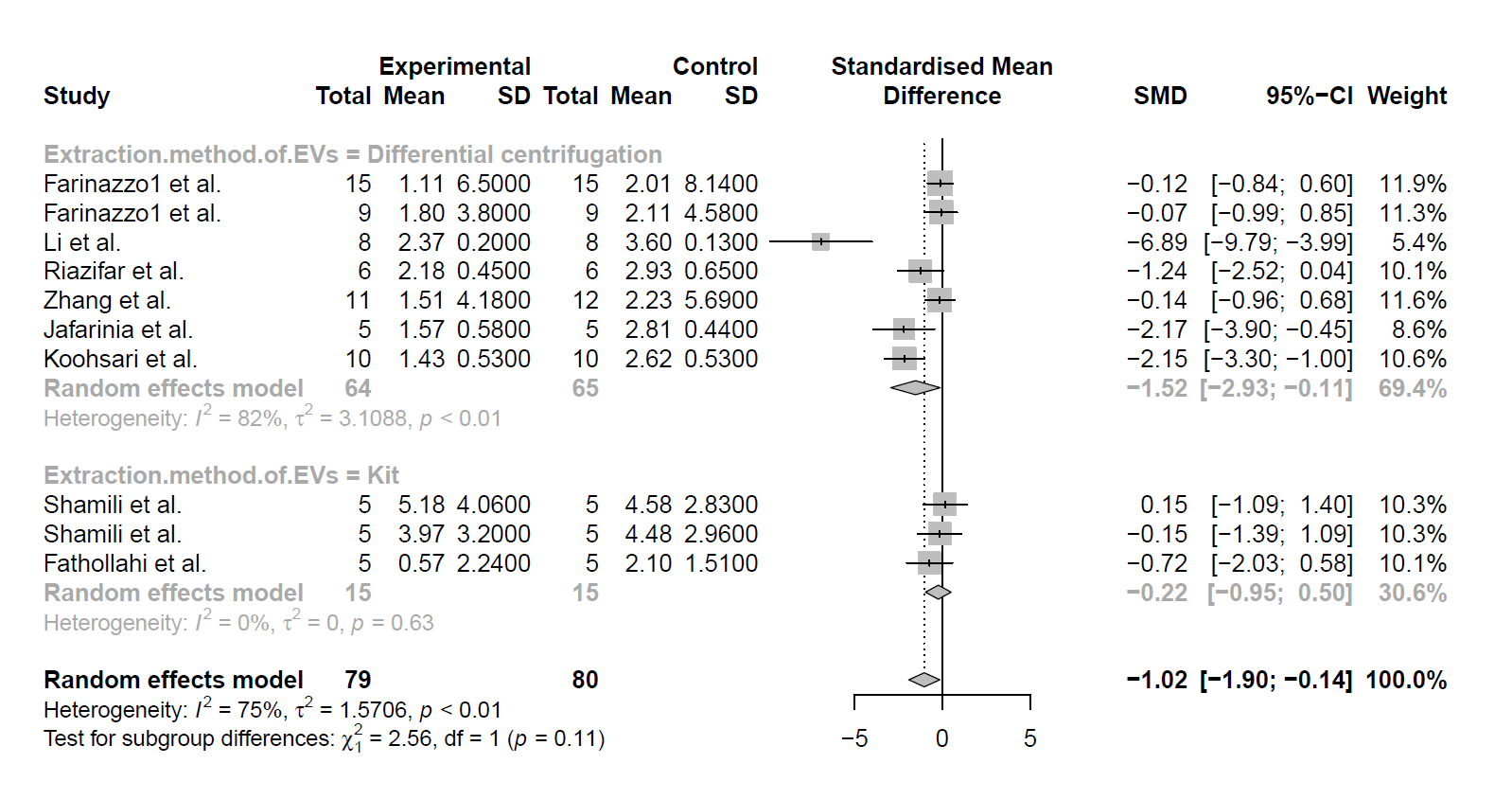
**

**Fig. S5. Subgroup analysis by the animal species of MS (rat versus mouse) for the clinical score.**

**
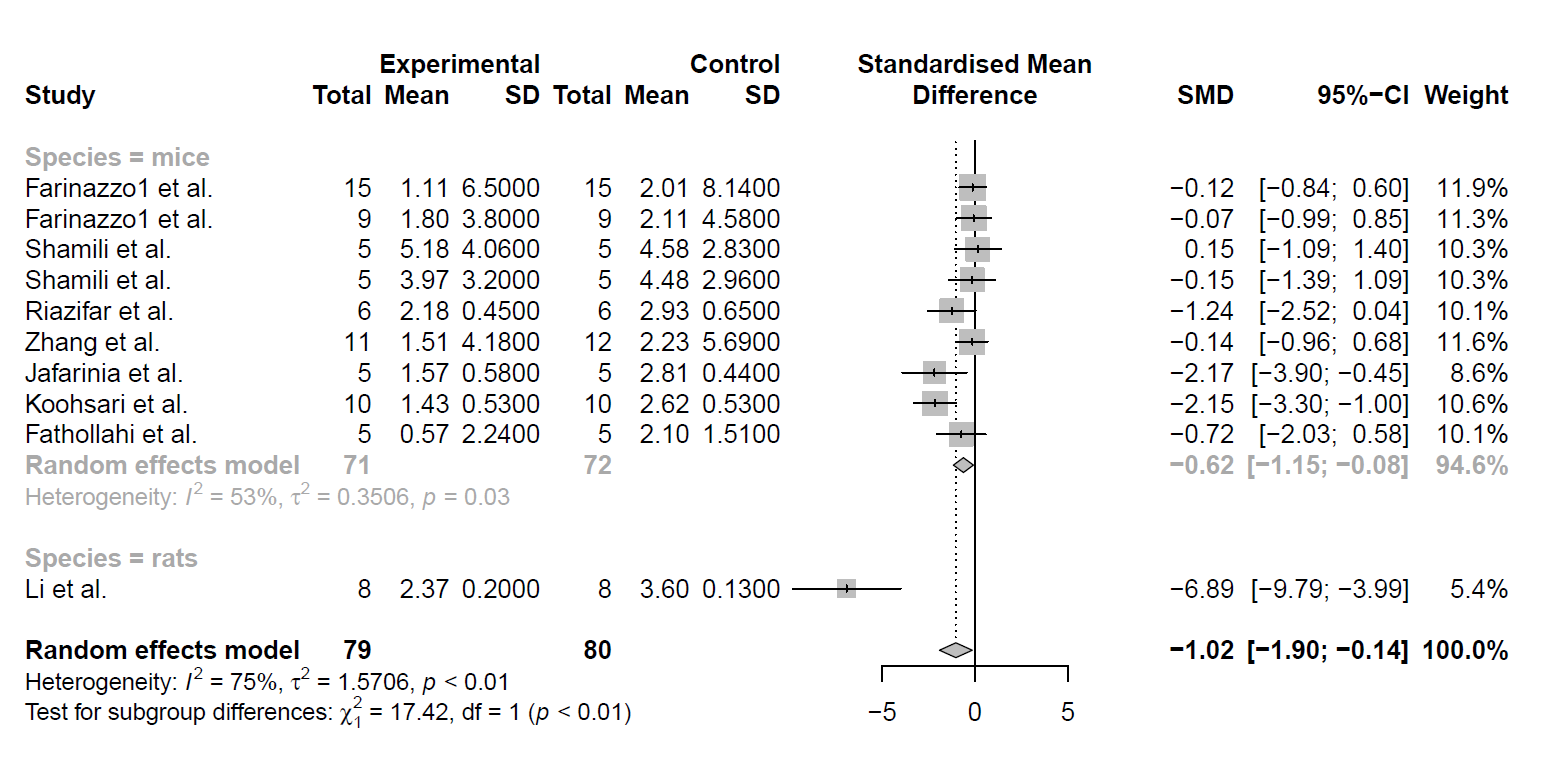
**

**Fig. S6. Subgroup analysis by the source of the MSCs (i.e. allogeneic, xenogenic, and syngeneic) for the clinical score.**

**
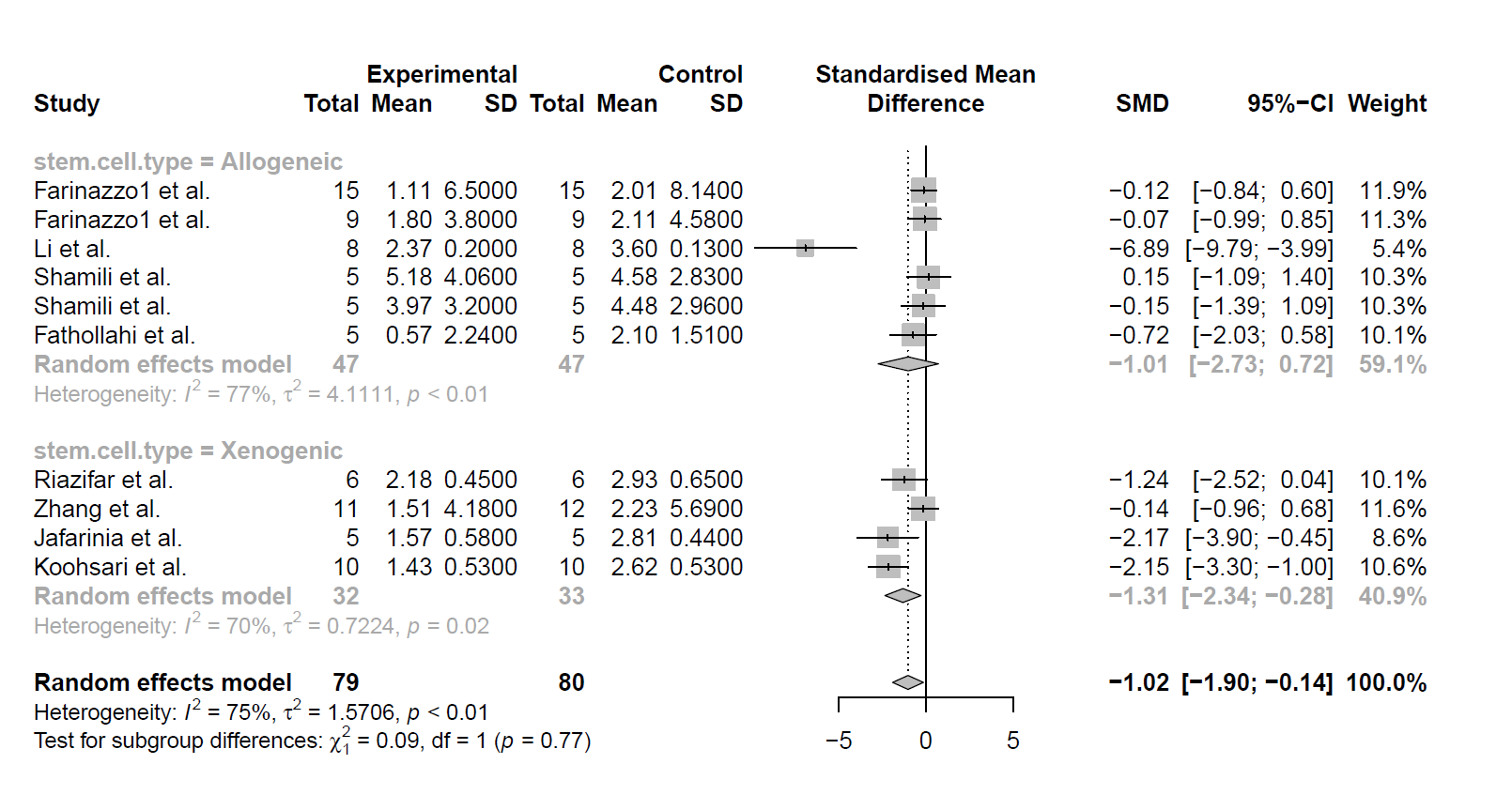
**

**Fig. S7. Subgroup analysis by the animal gender of MS for the clinical score.**

**
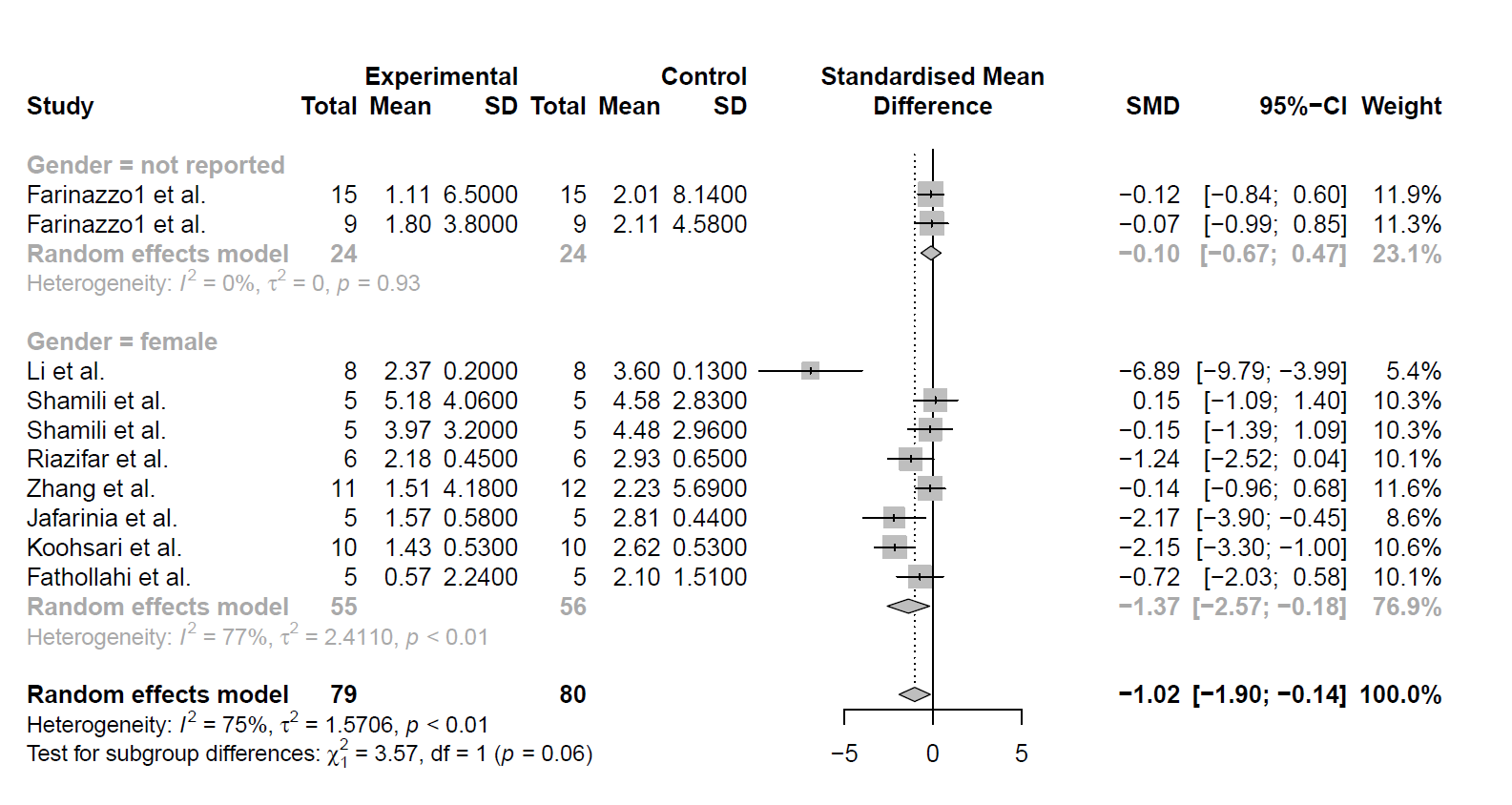
**

**Fig. S8. Subgroup analysis by the number of times of MS for the clinical score.**

**
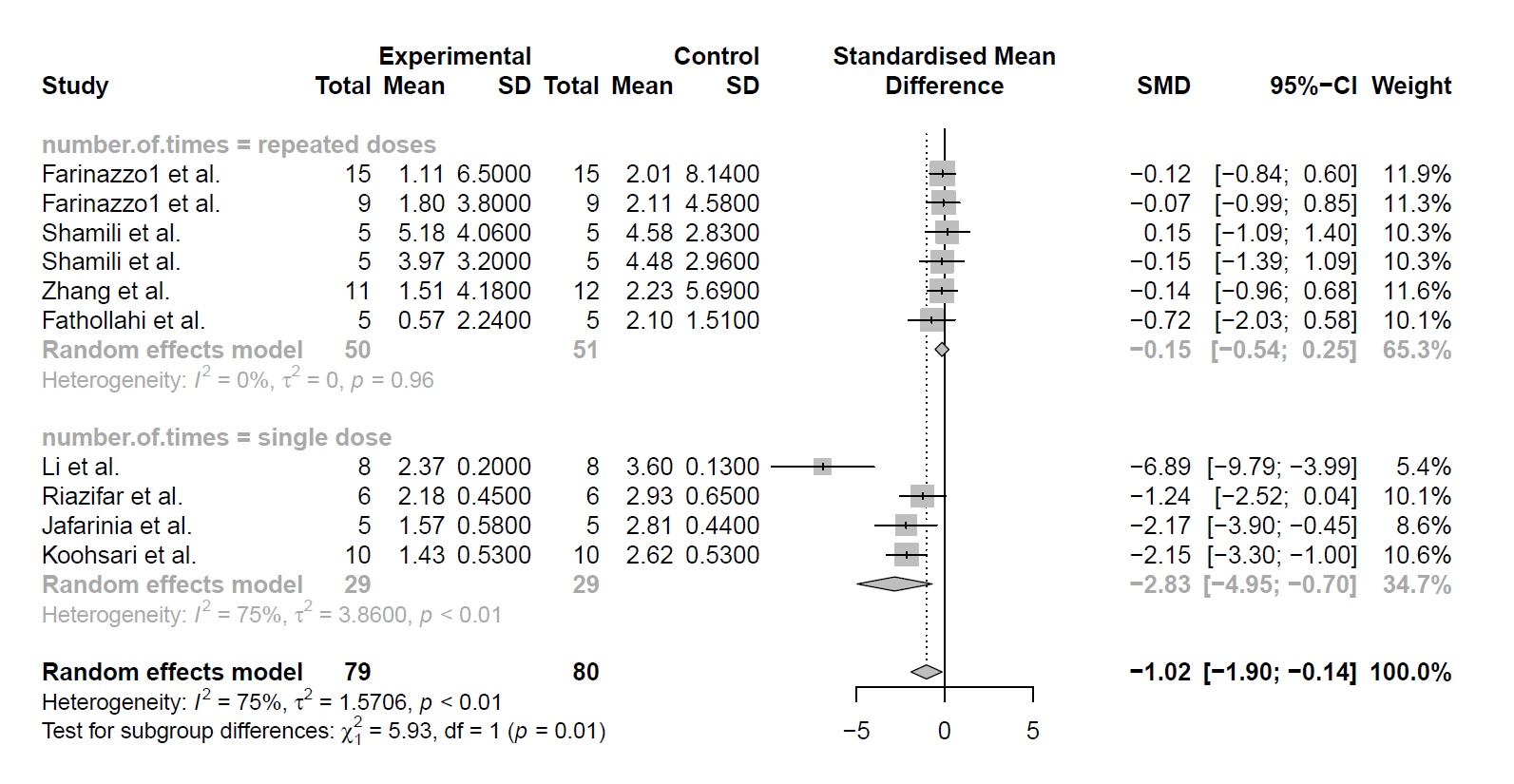
**

**Fig. S9. Subgroup analysis by the MSC-EVs dose of MS for the clinical score.**

**
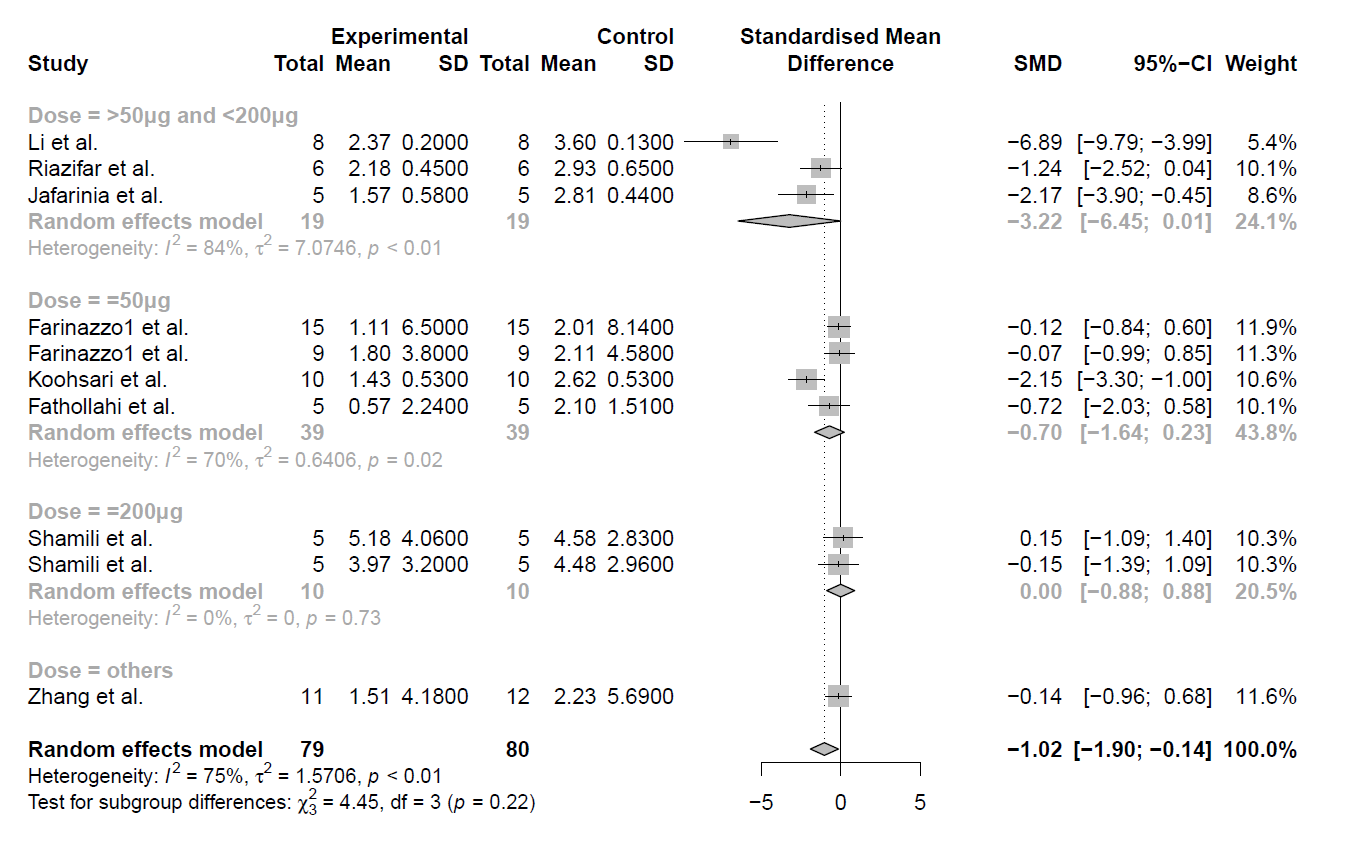
**
